# Evaluation of Trace Metal Levels in Tissues of Two Commercial Fish Species in Kapar and Mersing Coastal Waters, Peninsular Malaysia

**DOI:** 10.1155/2012/352309

**Published:** 2011-10-23

**Authors:** Fathi Alhashmi Bashir, Mohammad Shuhaimi-Othman, A. G. Mazlan

**Affiliations:** School of Environmental and Natural Resource Sciences, Faculty of Sciences and Technology, National University of Malaysia, Selangor, 43600 Bangi, Malaysia

## Abstract

This study is focused on evaluating the trace metal levels in water and tissues of two commercial fish species *Arius thalassinus* and *Pennahia anea* that were collected from Kapar and Mersing coastal waters. The concentrations of Fe, Zn, Al, As, Cd and Pb in these coastal waters and muscle, liver and gills tissues of the fishes were quantified. The relationship among the metal concentrations and the height and weight of the two species were also examined. Generally, the iron has the highest concentrations in both water and the fish species. However, Cd in both coastal waters showed high levels exceeding the international standards. The metal level concentration in the sample fishes are in the descending order livers > gills > muscles. A positive association between the trace metal concentrations and weight and length of the sample fishes was investigated. Fortunately the level of these metal concentrations in fish has not exceeded the permitted level of Malaysian and international standards.

## 1. Introduction

It is unfortunate that we, human beings without realizing the consequences of pollution, do a lot of activities that terribly ruin the nature, resulting in the denial of healthy environment to our successors. Water contamination is one of the serious concerns that affect the marine ecosystem with high concentration of trace level metals. Malaysia is one of the countries that critically face this issue since 1990. The reason for this alarming situation is due to the rapid economic growth that the country is experiencing for the past two decades. The contamination of water cannot be taken as price for this economic boom. 

According to Paquin et al. [1] the coastal or river waters are contaminated by the dumping of industrial wastages. The metals accumulated in these waters infect the humans by direct consumption of water or through consuming the affected organisms like fishes [2, 3] claim that when the level of trace metal concentrations exceeds the stipulated level, it turns out to be toxic. Very recently, the work in [4] has stated that the higher level of metal concentration will bring shattering effect to the ecological balance by altering the range of organisms in water. 

Several researchers, including [5–8], have studied the importance of fishes and their healthy benefits. They claim that fishes are the most healthy food with the high source of omega 3 fatty acids, that brings a lot of benefits to us, including the reduction of heart-related diseases. Apart from this, the fishes are rich source of vitamins, minerals, and proteins. Studies in [9, 10] reveal that 60 to 70% of protein needs are fulfilled by the consumption of fishes in Malaysia. But, [11–13] have analysed the other side of high fish consumptions. They claim that other than cardiovascular benefits, the exceeding level of fish diet brings negative impact to the human society.

Researches in [14, 15] reveal that iron and zinc are essential for the metabolism of fishes. At the same time, aluminium, cadmium, arsenic, and lead are added to the food chain of these organisms though they do not play any important role in the metabolic activities. Whereas [16] ascertain that when we consuming fishes with high accumulation of theses metals, over a long period of time, will bring harmful effects to us. Reilly and Barton [17, 18] added that the continual high dosage of Al consumption will result in lung fibrosis, osteomalacia, defective bone mineralization, dialysis dementia, and ferric-independent microcytic anaemia. Further studies regarding diseases related to high dose of mineral consumption can be summarized as follows. High Cd Accumulation brings skeletal damage, kidney dysfunction, and reproductive deficiencies [19]; cardiovascular disease, skin disorders, cancer, and neurotoxicity are triggered by arsenic consumption [20]; Pb, termed as neurotoxins, brings cardiovascular diseases to adults and reduced mental development in children [19, 20]. 

According to Canli and Atli [21] Fe and Zn are very vital for the normal metabolism for the schools of fishes. At the same time iron is one of the important trace metals that highly benefits humans. It serves as the oxygen conductor between the tissues and lungs. Camara et al. [22] have established the health benefits of advocated level of mineral consumption. They claim that deficiency of Zn will cause loss of appetite, growth retardation, skin changes, and immunological abnormalities. But Tüzen [23] has stated that though Zn has biological significance, excessive consumption of these kinds of metals will affect the humans. The trace metal sewage from industries pollutes water and fishes in turn. The consumption of the affected fishes over a prolonged period will harm the health of humans. 

Fortunately previous studies reveal that the trace metal concentration level in fishes is not that much alarming in South East Asian countries. The researchers have examined muscles, livers, and gills of fishes as these organs play different roles in bioaccumulation process [24]. Hamilton and Mehrle [25] say that the concentration level of metals in gills represents the level of metals in water, where they dwell. The concentrations of metals in liver represent their storage level. Metallothioneins (MTs) are the metal-binding proteins accumulated in livers, whereas [26, 27] assert that metal accumulation in the muscles of fishes is dangerous as they are the most edible part. They also established that, environmental evaluation in aquaecology shall be conducted in water, organisms, or sediments. Each of these components provides partial image of metal accumulation in the whole ecosystem. 

According to Marcovecchio and Moreno [28], studying the trace level in organisms reflects the real degree of pollution in the related environment. References [29–31] state that generally fishes are used as the medium for monitoring anthropogenic pollution level in the environment. As fishes are the last level of the food chain, the polluted varieties will easily pass the metals into the humans when they are consumed [32–35].

The marine ecosystem of Kapar is selected as it is located in the Strait of Malacca which consists of many pollution sources situated around it. This locality gets infected by a great variety of pollutants due to the existence of large number of international shipping lanes and the concentration of agriculture, industrialization, and urbanization activities along the coast of Peninsular Malaysia [36]. Moreover, the Strait of Malacca is one of the most vulnerable areas to contamination by oil spills [37]. On the other hand, the Strait of Malacca is the most important fishing ground in Malaysia, accounting for approximately 70% of total fish landings of the country [37]. 

Apart from the above-mentioned sources of pollution, an electric power station that uses coal and discharges the polluted, preused water into the surface water systems surrounding Kapar, also contribute to the trace metal pollution of the marine ecosystem in this area. Moreover, Kapar has great importance for the local fishery industry therefore, it is vital to estimate the selected metals in fish of the Kapar coastal water, to define the current trace metal levels in the fish as well as to monitor the trends of change in fish trace metal levels with time. 

However, evaluation of the levels of the same metals in Mersing allows for comparison between the two areas, particularly in terms of the kinds and effects of different pollution sources in the two areas. This study is focused on measuring the concentration level of selected metals (Al, Fe, Zn, As, Cd, and Pb) in the muscles, livers, and gills of selected species of fishes and the pollution level in the coastal areas in Malaysia. Moreover, the relationships between the trace metal levels in these tissues and the length and weight of fishes were also investigated.

## 2. Materials and Methods

### 2.1. Reagents

The reagents with suprapur quality, analytical grade Nitric acid (65%), and hydrogen peroxide (30%) were acquired from Merck (Darmstadt, Germany) along with the stock standard solutions of Al, Fe, Zn, As, Cd, and Pb in concentrations of 1,000 mg/L. Prior to the experiments the apparatus were sterilised by soaking them overnight in diluted nitric acid (10%) and were later rinsed with deionised water. The experiments were conducted using the distilled deionised water. 

### 2.2. Apparatus

In this study we have used Perkin Elmer model Elan 9000 inductively coupled plasma mass spectrometry (ICP-MS, USA), [40]. After calibrating the instrument with standard solutions derived from commercial materials, it was optimized according to the manufacturing standards. Besides these initiations, the cones and tubes were thoroughly cleaned to get rid of any possible residues.  Table 1 shows the analytical conditions for determining the trace metals by the inductively coupled plasma mass spectrometry (ICP-MS).

### 2.3. Study Area

**Figure 1:**
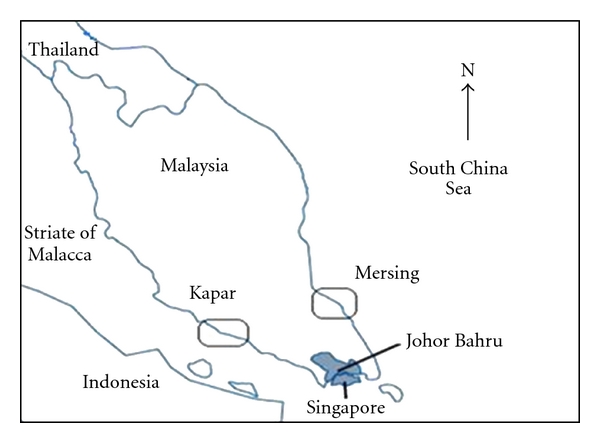
Locations of the sampling sites.

Water and fish sampling were done at two different stations of coastal waters of Peninsular Malaysia in October 2009. Stations shown on the map (Figure 1) were chosen in relation to the contamination gradient.

The first station chosen was Kapar (3°11′54′′ N, 101°32′66′′ E) located in Selangor on the west coast of Peninsular Malaysia near the Sultan Salahuddin Abdul Aziz Power Plant station. In terms of pollution, the water quality of Kapar coast is influenced by various industrial outputs, discharged directly to the sea or by rivers. The second station was Mersing (2°25′60′′ N, 103°49′60′′ E) in Johor on the west coast of Peninsular Malaysia, it is relatively clean when compared with Kapar. 

### 2.4. Samples Collection and Samples Preparation

#### 2.4.1. Collection of Water Samples

For research purpose a stint of 200 ml of water was collected with the help of 21 cc capacity automated sampler. The sample was collected from the surface of the coastal water (depth range < 10 centimetre). The sample was then filtered using Whatman 0.45 *μ*m membrane filter paper, and filled in polyethylene bottles (amber coloured). These bottles were pre washed with 1 (N) HNO_3_ and deionised water. Later 3 mL of concentrated HNO_3_ was added to the collected sample to avoid oxidation and preserved at 4°C, prior to analysis.

#### 2.4.2. Collection of Fish Samples

Two commercially significant and nutritious fish species, namely, duri (*Arius thalassinus*) and gelama (*Pennahia anea*), were selected and collected with various fishing methods by fishermen (Table 2). Ten fresh fish specimens of both the species, from each station, were collected from local fishermen. The samples were stored in a cool box (−4°C) and transported to the laboratory for metal analysis. Total length (cm) and weight (g) of the fish samples were measured before dissection.

The specimens were dissected with sterilized stainless steel equipment. The dissected parts such as muscle, liver, and gills were later dried in an oven at 80°C until constant weight was obtained. The homogenized samples (muscle, liver, and gills) were digested in triplicate in a microwave oven digestive system (Start D Microwave Digestive System) with HNO_3_ (65% Merck) and H_2_O_2_ (30% Merck) in Teflon vessels. The residues were filtered through 0.45 *μ*m Whatman filter paper (Whatman international Ltd. Cat) and transferred to a 50 mL volumetric flask and diluted to level with deionised water in the case of muscle and gills. However, in the case of liver tissues, the final dilution volume was 25 mL rather than 50 mL [41]. 

Analytical blanks were run in the same way as the samples and determined using standard solutions, prepared in the same acid matrix. All chemical materials and standard solutions used in this study were obtained from Merck and were of analytical grade. 

### 2.5. Analysis of Metals

As discussed earlier in Section 2.2, the concentrations of iron (Fe), zinc (Zn), aluminium (Al), arsenic (As), cadmium (Cd), and lead (Pb), in water and two species of fish, were examined using the inductively coupled plasma mass spectrometry. The analytical findings were articulated in terms of micrograms of metal in every gram of fish on dry weight basis (*μ*g/g dry weight). The performance assessment of this method was done by examining a standard reference material of marine biota sample (SRM2976, freeze-dried mussel tissue, National Institute of Standards and Technology, USA).

### 2.6. Statistical Analysis

Due to the lack of normal distribution of data, the log transformation was implemented for the normalization process. To examine the vital differences in the concentrations of heavy metals in the two research sites, the *t*-test was conducted. Moreover, to investigate the denoting dissimilarity of concentrations of trace metals among the three fish organs, the Kruskal-Wallis test was used. Pearson rank correlation analysis was employed, to measure the latent associations of metal concentrations with fish weight and length. For which a *P *value less than 0.05 was considered as suggestive of statistical significance. SPSS for windows, version 16.0 was used to perform all the above-mentioned tests.

## 3. Results and Discussion

### 3.1. Validation of Analytical Methods

The precision and accuracy of the applied analytical method was validated by accurate analysis of standard reference material of marine biota sample (SRM2976, freeze-dried mussel tissue, National Institute of Standards and Technology, USA). All the runs were carried out in triplicate. The results obtained on the SRMs are showed in Table 3 which was in a good agreement with the certified values for all metals. Recovered values of all metals were between 83% and 109% of the certified value.

#### 3.1.1. Quality Control

It is vital for the analytical instruments (ICP-MS) to meet the standard before it can produce a reliable data. Calibration curve of each element must be able to produce good correlation coefficient *r* ^2^ = 0.999.

#### 3.1.2. Instrument Detection Limit (IDL)

The IDL is the smallest signal that can be differentiated from background noise by a specific device. The method detection limit should be always higher than the IDL, whereas the IDL is thrice equal to the standard deviation of 10 replicates measurements of calibration blanks signal at the selected elements.

#### 3.1.3. Limit of Detection (LOD)

The LOD is the least amount of a substance that can be distinguished from the absence of it (a blank value) within a stated confidence limit (generally 1%). The method detection limit is defined as the concentration corresponding thrice to the standard deviation of ten reagent blanks [42].  Table 4 shows the method detection limit (*μ*g/g) of five metals and the FDA recommended health-criteria concentrations (*μ*g/g) of five metals in seafood [38, 39]. The detection limit values were found to be 0.287 *μ*g/g for Al, 0.492 *μ*g/g for Fe, 0.474 *μ*g/g for Zn, 0.403 *μ*g/g for As, 0.193 *μ*g/g for Cd, and 0.606 *μ*g/g for Pb which were much lower than the recommended health-criteria values.

#### 3.1.4. Limit of Quantitation (LOQ)

The LOQ is mathematically expressed as equal to 10 times the standard deviation of the results for a sequence of replicates used to establish a reasonable boundary of detection. The LOQ values were found to be 2.87 for Al, 4.92 *μ*g/g for Fe, 4.73 *μ*g/g for Zn, 4.03 *μ*g/g for As, 1.95 *μ*g/g for Cd, and 6.16 *μ*g/g for Pb.

### 3.2. Trace Metals Contents in Water

Analysis on water quality of baseline study for Kapar and Mersing seawater are necessary to predict the level of pollutant as well as to the environment in the study areas.  Table 5 shows the water temperature, pH, dissolved oxygen (DO), and the trace metal levels in the seawater samples from Kapar and Mersing. The water temperature from Mersing and Kapar ranges from 19.6 to 22.5°C; the variation in water temperature was mainly due to prevailing weather conditions. The statistical analysis showed that there was no significant difference between the two locations (*P* > 0.05). Moreover, the pH has ranged between 7.23–7.56. The standard pH for seawater is 6.5–8.5 [43], and the values obtained were within the recommended standard, and there was no significant difference in pH for the two sampling sites. The lowest dissolved oxygen (DO) value recorded was 4.37 mg/L at Kapar, while the highest was 7.90 mg/L at Mersing. Generally, the dissolved oxygen will be affected by water temperature, tides, and depths. Furthermore, the maximum concentration of the metals in the water samples in the descending order were Fe > Al > As > Zn > Pb > Cd and Fe > As > Cd > Zn > Al > Pb from Kapar and Mersing, respectively. Al, Zn, and As had higher concentration in Kapar, whereas the Fe and Cd had higher in Mersing. There was no significant difference in metal concentrations in the two sampling locations. Additionally, the comparison of trace metals level with NWQSM and WHO [44, 45] showed that all the metal concentrations were below the maximum acceptable concentration (MAC), except for Cd from both sites that showed high levels exceeding the international standards suggesting that adverse effects to aquatic organisms would frequently occur.

### 3.3. Trace Metals Contents in Various Organs in Fish

Knowledge about heavy metals concentration in fish is important with respect to nature management and human consumption. Levels of six metals (*μ*g/g dry wt.) in muscle, liver, and gill tissues of two fish species collected from the coastal waters around Kapar and Mersing are shown in Tables 6 and 7. Generally, the highest concentrations of iron, zinc, aluminium, arsenic, and lead were found in the liver tissues of both examined fish species. The analysis of variance proved that the mean concentrations of metals in the organs of each species were significantly different (*P* < 0.05) in both the species except for Cd. The concentrations of the studied metals decreased in the following order Fe > Zn > Al > As > Pb > Cd in the two species. Iron exhibited the highest concentrations in all the examined organs of both species, followed by Zn. On the other hand, the levels of Pb and Cd were generally the lowest. Similar findings were reported by many researchers [14, 46–48]. 

It is observed that Fe concentration was the highest in both species and both study areas. In the present study, with the exception of Al, liver had significantly higher trace element concentrations than gills and muscle. It is observed that the mean concentrations of metals in the muscle, liver, and gills of each fish species showed great variations, this may be related to the differences in ecological needs, swimming behaviours, and the metabolic activities among different fish species. 

The differences in metal concentrations of the tissues might be due to their capacity to induce metal-binding proteins such as metallothioneins. Our study showed that the metal levels in liver and gills were highest in the sampled species. It is well known that large amount of metallothioneins induction occurs in the liver tissue of fishes. The adsorption of metals onto gill surface could also be an important influence in total metal levels of the gill [30]. 

The mean concentrations of Fe in the muscles of *P. Anea* and *A. thalassinus *in Kapar were 34.91 *μ*g/g and 53.84 *μ*g/g, respectively. However, in Mersing the mean concentrations were 21.47 *μ*g/g in *P. Anea* and 21.62 *μ*g/g in *A. thalassinus*. It is revealed that Fe concentrations varied significantly (*P* < 0.05) between the two stations. Higher Fe concentration in muscles of both species was found in the fish from Kapar than that of Mersing. The reason for this is that the Kapar area is polluted by various sources such as electrical power station, international shipping activities, and urban and agricultural activities. Similarly, the concentrations of Fe in the liver tissues of *P*. *anea* and *A. thalassinus *in Kapar were, approximately, 1976.0 *μ*g/g and 1008.0 *μ*g/g, respectively. But in the same tissues of the fish from Mersing the concentrations were 526.0 *μ*g/g and 924.6 *μ*g/g, respectively. The levels of iron in the muscles of Mediterranean Sea fish that are reported in the literature ranges from 59.6 and 73.4 *μ*g/g [31]. The concentrations of Fe in the fish muscle were reported to have the range of 24.1–50.3 *μ*g/g in Parangipettai Coast, India [49] and the range of 49.9–889 *μ*g/g in the Turkish seas [47]. Therefore, the levels of iron in the fish muscles reported in this study are generally in accordance with the literature.

According to the results (Tables 6 and 7), concentrations of Zn in the livers of *P*. *anea* and *A. thalassinus *collected from Kapar and Mersing were 114.1 *μ*g/g and 555.9 *μ*g/g (Table 6), and 104.8 *μ*g/g and 341.9 *μ*g/g (Table 7), respectively. Generally, high concentrations of Zn were observed in the livers of *A. thalassinus *in both the studied areas. The mean concentrations of Zn in the muscle tissues of *P. Anea* and *A. thalassinus *collected from Kapar were around 26.3 *μ*g/g and 51.0 *μ*g/g, respectively. However, in Mersing the respective concentrations were 18.1 *μ*g/g and 25.4 *μ*g/g. Higher Zn concentrations in the muscle tissues of both species were found in Kapar than in Mersing. 

The observed differences can be explained by the fact that the concentrations of these metals depend to a great extent on species, sex, biological cycle, and on the part of the fish analyzed [23]. Moreover, ecological factors such as season, location/environment of development, nutrient availability, and temperature and salinity of the water, may contribute to variations in the metal concentrations in fishes. Ranges of Zn concentrations reported earlier in the muscles and livers of Malaysian marine fish were 15.4–60.1 *μ*g/g and 27.1–95.3 *μ*g/g, respectively [40]. Another study conducted in Langkawi Island showed that all species had higher concentrations of Zn than of other metals and that the concentrations in muscles ranged from 34.3 *μ*g/g to 49.4 *μ*g/g [50]. Accordingly, the Zn concentrations in the fish muscles detected by the present study are similar to those reported by [50].

In this study, the concentrations of aluminium were the highest in the gills and it ranged from 13.7 *μ*g/g in *P. anea *to 538.6 *μ*g/g in *A. thalassinus *in Kapar, and from 91.0 *μ*g/g in* P.anea *to 850.14 *μ*g/g in *A. thalassinus *at Mersing, whereas in *A. thalassinus*, the Al concentration was 7.2 *μ*g/g in muscle and 28.4 *μ*g/g in liver at Kapar station and it was 5.4 *μ*g/g and 10.0 *μ*g/g in the fish muscle and liver tissues, respectively, at Mersing station.

On the other hand, the concentrations of Al in the muscle and liver tissues of *P. anea* was 3.0 *μ*g/g and 13.7 *μ*g/g, respectively, in Kapar while the respective concentrations in Mersing were 1.5 *μ*g/g and 5.1 *μ*g/g, respectively. The mean Al concentrations were higher in the three organs fish species captured from Kapar than its concentration in the same organs of the fish species collected from Mersing except gills of *A. thalassinus* from Mersing. The Al concentrations were reported to fall within the range 1.50–4.50 *μ*g/g in fish muscles from the Parangipettai Coast, India [49]. On the other hand, the Al concentrations were reported earlier to fall in the range of 51.9–166.3 *μ*g/g in muscles and in the range of 229.01–1412.7 *μ*g/g in gills of Malaysian marine fish from Kapar [51]. As such, the Al concentrations observed in this study generally correspond with the values reported in the literature.

Arsenic levels in the muscles of the analyzed fish ranged from 3.8 *μ*g/g in *P. anea* in Kapar to 14.2 *μ*g/g in *A. thalassinus* in Mersing. Whereas, the arsenic levels in fish livers ranged from 11.8 *μ*g/g in *P. anea* from Kapar to 21.9 *μ*g/g in *A. thalassinus* from Mersing. The arsenic levels in the fish gills ranged from 4.9 *μ*g/g in *P. anea* from Mersing to 13.6 *μ*g/g in *A. thalassinus* from Kapar (Tables 6 and 7). Unfortunately we do not have sufficient data of arsenic levels in fish tissues from Malaysia, to be compared with our findings.

According to published literature, ranges of arsenic concentrations reported earlier in the muscles of Malaysian marine fish were 1.05–2.14 *μ*g/g [52]. Another study conducted showed that arsenic content of fish from Indian coastal waters was within the range of 0.01–0.63 *μ*g/g [53] which are well below the arsenic levels detected in fish tissues by this study.

In this study, the lead concentrations in muscles ranged from 0.1 *μ*g/g in *P. anea* in Kapar to 0.2 *μ*g/g in *A. thalassinus* in Mersing. While in the livers the concentrations ranged from 1.1 *μ*g/g in *P. anea *from Mersing to 1.5 *μ*g/g in *A. thalassinus* in Kapar. On the other hand, the concentration ranged in gills from 1.96 *μ*g/g in *P. anea *in Mersing to 2.03 *μ*g/g in *A. thalassinus* in Kapar. Lead levels reported earlier in the literature fall in the range of 0.018–0.023 *μ*g/g for muscles and 0.115–0.380 *μ*g/g for livers of fish from Mersing. Moreover, the lead level ranged from 0.026–0.72 *μ*g/g in muscles and 0.041–0.872 *μ*g/g in livers of fishes from Langkawi coastal waters of Malaysia [40]. Hence, the Pb concentrations reported herein comply with these ranges.

The results of this investigation reveal that there is no significant variation in Cd levels (*P* > 0.05). The highest concentrations were observed in the livers of the fish species from Mersing, where the mean Cd concentration ranged from 2.075 *μ*g/g in livers of *A. thalassinus* to 2.458 *μ*g/g in livers of *P. anea *from Mersing coastal water. While the highest levels of Cd in the muscles were recorded as 0.088*μ*g/g in *A. thalassinus* from Kapar, in contrast, the lowest value of Cd detected was 0.021 *μ*g/g in the muscles of *P. anea *from Mersing, whereas the Cd levels reported in the literature fall in the range of 0.14–0.57 mg/kg and 0.15–0.52 mg/kg for muscles of *A. thalassinus* and *P. anea *from the same study area Kapar [51]. Another study was conducted on commercial marine fish from Klang Valley, Malaysia, which concluded that the mean Cd concentrations in the fish muscles ranged from 0.121 mg/kg to 1.594 mg/kg [54]. The third study investigated the marine fin fish captured from the coast of Langkawi Island in Malaysia and reported that the mean Cd concentrations in the fish muscles ranged from 0.30 *μ*g/g to 0.90 *μ*g/g [50]. Compared with the literature from different Malaysian marine coastal waters, our results for Cd concentration is lower than the literature. Cd and Pb have higher tendencies to bioaccumulate in the fish liver tissues which involves in the detoxification process. The presence of free protein-thiol group content and metallothioneins binding proteins in the liver forms a strong fixation with the heavy metals [55]. Meanwhile fish liver acts as major site for homeostasis [56].

The variability in heavy metal levels in different species depends on feeding habits [26], ecological needs, and metabolism [30], age, size, and length of the fish [57], and fish habitat [21]. Concentrations of trace metals detected in the muscle, gill, and liver samples indicate different bioaccumulation potentials. Muscles seem to be a transitory tissue in the pathway of metal uptake and in metal storage, whereas the liver appears to be the tissue, specialized in metal storage and detoxification [58]. The gills comprise the chief exposure tissue and early uptake site of the soluble, waterborne metals in which metal concentrations are the highest in the early stages of exposure, before these metals are transported to other fish tissues [59]. Although human activity is concentrated in the west coast of Peninsular Malaysia, compared with the east coast, there is some contamination with heavy metals in the east coast. The results of the present study suggest that, at some point, sources of heavy metal contaminations are present in the east coast of Peninsular Malaysia in spite of the relatively low human activities.

**Figure 2:**
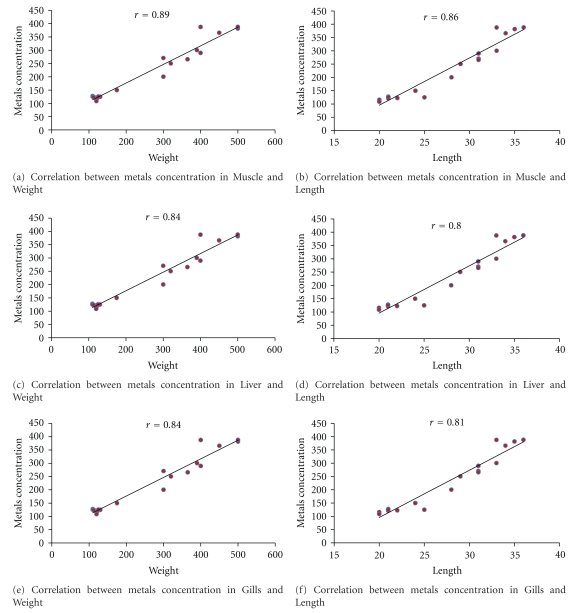
Correlation analysis.

The relationships between body size and trace element concentrations in the two fish species were also investigated and significant positive correlations between the total fish length and weight and heavy metal concentrations were found (*P* < 0.05) (Figure 2). Particularly, the concentrations of some of the heavy metals of concern had positive, high correlations with fish weight and total length. On the other side, metal concentrations were more affected by fish weight than by length. Our findings were in agreement with results reported by [60].

## 4. Conclusion

This study was undertaken to provide information on trace metal concentrations in water and two fish species from Kapar and Mersing. The highest metal concentrations were found in the fish liver and gill tissues, while the muscles tend to accumulate relatively low metal levels. Generally, the Fe concentrations were the highest in water samples and all organs of the two species in both study areas except the muscles of *P. anea* from Kapar which had higher Zn concentration, and the gills of *A. thalassinus* from both Kapar and Mersing had higher Al and Zn levels, respectively. Moreover, the *A. thalassinus* species had higher metal concentrations than the *P. anea*. In water samples, Cd concentrations in both sites exceed the international standards, but still in the permissible levels of national standards. The mean concentrations of heavy metals, analyzed in the muscles of both species, were lower than the maximum concentrations recommended by [61–63]. The concentrations of Al, Fe, Zn, As, Cd, and Pb in muscle tissues should pose no acute toxicological risks to human health. This study revealed that the studied metals concentrations are generally low in the tissues of the examined fish in the two study areas. Although the levels of these heavy metals are not high, a potential danger may emerge in the future depending on pollution sources. The data may be taken as a convenient base line against which any future pollution trends can be evaluated.

## Figures and Tables

**Table 1:** ICP-MS operating conditions and performance.

Performance	Operating condition
RF Generator	40 MHz
RF Power	1000 W
Spray Chamber	Ryton Scott
Nebulizer	Cross-Flow
Plasma gas flow	15.0 L/min
Auxiliary gas flow	1.0 L/min
Nebulizer gas flow	0.60 L/min
Sampler & skimmer cone	Nickel
Sweeps/Reading	20
Reading/Replicates	3

**Table 2:** The fish samples and the average length and weight of the species examined in present study.

Species	Samples number	Family	Common name	Habitat	Total weight (g)	Total length (cm)
*A. thalassinus*	10	Ariidae	Sea catfish	Demersal	300–500	31–36
*P. anea*	10	Sciaenidae	Big eye Croaker	Demersal	112–130	21–25

**Table 3:** Observed and certified^(1)^ values of elemental metal concentrations (*μ*g/g dry weight).

Element	Certified value	Measured value	SRD%	Recovery (%)
Al	134 ± 34	128.23	0.2	96
Fe	158 ± 8	144.64	1	92
Zn	137 ± 13	115.20	1.1	84
As	13.3 ± 1.8	14.452	2.5	109
Cd	0.82 ± 0.16	0.679	12.5	83
Pb	1.19 ± 0.18	1.026	0.4	86

^(1)^Certified mussel standard reference material (SRM) 2976.

**Table 4:** Method detection limits of trace metals.

Trace metals	Detection limit (*μ*g/g)	*Health-criteria levels (*μ*g/g)
Al	0.287	—
Fe	0.492	—
Zn	0.474	480
As	0.403	86
Cd	0.193	4
Pb	0.606	2

*FDA recommended health criteria concentrations (*μ*g/g), [38, 39].

**Table 5:** Trace metal concentrations (mg/L), water temperature (T), pH, dissolved oxygen (DO mg/L) in sea water from Kapar and Mersing.

Location				Metals					Parameters	
		Al	Fe	Zn	As	Pb	Cd	T (C°)	pH	DO (mg/L)
Kapar	Mean	0.048	0.33	0.021	0.036	0.010	0.010	23.05	7.72	5.66
	Max	0.049	0.34	0.033	0.040	0.014	0.011	24.80	7.65	5.62
	Min	0.047	0.32	0.015	0.020	0.020	0.010	21.30	7.79	5.70

Mersing	Mean	0.012	0.36	0.015	0.030	0.010	0.019	21.25	7.28	6.73
	Max	0.013	0.40	0.016	0.033	0.014	0.030	23.60	7.52	6.60
	Min	0.011	0.32	0.014	0.028	0.002	0.011	18.90	7.64	6.86

NWQSM*		0.5	1	0.5	0.1	0.5	0.01	—	—	5–7
WHO**		—	—	5	0.01	0.01	0.003	—	6.5–8.5	—

NWQSM* National Water Quality Standards for Malaysia. WHO**World Health Organization.

**Table 6:** Concentrations (*μg* *g* ^−1^ dry wt) of heavy metals (mean ± SD) in different organs of fish collected from the coastal waters of Kapar, Malaysia.

Species	Organ			Elements			
		Al	Fe	Zn	As	Cd	Pb
*A. thalassinus*	Muscle	7.24 ± 0.0	53.84 ± 5.1	50.99 ± 5.34	12.58 ± 0.8	0.088 ± 0.01	0.12 ± 0.01
	Liver	28.38 ± 1.92	1007.1 ± 11.7	550.89 ± 6.75	14.17 ± 1.03	1.01 ± 0.07	1.54 ± 0.19
	Gills	538.6 ± 3.48	805.6 ± 3.38	840.89 ± 5.2	13.59 ± 0.69	0.048 ± 0.01	2.03 ± 0.05

*P. anea*	Muscle	3.00 ± 0.11	21.62 ± 4.7	26.32 ± 1.6	3.28 ± 0.65	0.048 ± 0.01	0.13 ± 0.004
	Liver	13.72 ± 1.12	1975.0 ± 38.9	114.11 ± 2.16	11.75 ± 0.13	0.694 ± 0.05	0.57 ± 0.03
	Gills	299.5 ± 0.75	891.6 ± 28.6	60.21 ± 0.44	4.75 ± 0.65	0.690 ± 0.05	0.26 ± 0.02

WHO*		—	50	150	0.02	0.2	0.2
FAO**		—	—	30–100	7.88^a^	0.2	0.5–0.6
MFR***		—	—	100	—	1	2

*WHO (1989), **FAO (1992), ^a^FAO 1983, ***Malaysian Food regulation (1985).

**Table 7:** Concentration (*μg* *g* ^−1^ dry wt) of heavy metals (mean ± SD) in different organs of fish collected from the Mersing coastal waters, Johor Bahru, Malaysia.

Species	Organ			Elements			
		Al	Fe	Zn	As	Cd	Pb
*A. thalassinus*	Muscle	5.44 ± 1.25	34.91 ± 1.74	25.39 ± 0.71	14.2 ± 2.34	0.02 ± 0.03	0.2 ± 0.02
	Liver	10.05 ± 1.7	924.6 ± 24.7	341.9 ± 3.35	21.89 ± 0.9	2.075 ± 0.1	0.87 ± 0.07
	Gills	850.1 ± 7.1	822.76 ± 9.9	246.55 ± 7.4	7.65 ± 0.1	0.02 ± 0.01	0.24 ± 0.02

*P. anea*	Muscle	1.46 ± 13	21.47 ± 1.86	18.1 ± 0.79	3.76 ± 0.2	0.023 ± 0.02	0.17 ± 0.05
	Liver	5.13 ± 0.65	525.96 ± 17	104.84 ± 1.23	8.38 ± 0.22	2.46 ± 0.02	1.07 ± 0.07
	Gills	91.03 ± 2.7	461.4 ± 8.6	66.24 ± 1.3	4.88 ± 0.13	0.05 ± 0.01	1.96 ± 0.16

WHO*		—	50	150	0.02	0.2	0.2
FAO**		—	—	30–100	7.88^a^	0.2	0.5–0.6
MFR***		—	—	100	—	1	2

*WHO (1989), **FAO (1992), ^a^FAO 1983, ***Malaysian Food regulation (1985).

## Abbreviations

cm: Centimeter
g: Gram
*μ*g/g: Micrograms per gram
*μ*g/L: Micrograms per liter
Mg/L: Milligrams per liter
SD: Standard deviation
RSD: Relative standard deviation.
